# Influence of sugammadex versus neostigmine for neuromuscular block reversal on the incidence of postoperative pulmonary complications: a meta-analysis of randomized controlled trials

**DOI:** 10.1186/s13741-021-00203-6

**Published:** 2021-09-20

**Authors:** Jia-Feng Wang, Zhen-Zhen Zhao, Zheng-Yu Jiang, Hui-Xing Liu, Xiao-Ming Deng

**Affiliations:** 1 Faculty of Anesthesiology, Changhai Hospital, Naval Medical University, 168 Changhai Road, Shanghai, 200433 China; 2 Department of Clinical Epidemiology and Biostatistics, Peking University People’s Hospital, No. 11 Xizhimen South Street, Xicheng District, Beijing, 100044 China

**Keywords:** Neuromuscular block, Postoperative pulmonary complications, Sugammadex, Neostigmine, Meta-analysis

## Abstract

**Background:**

The influence of sugammadex for reversal of neuromuscular block (NMB) on postoperative pulmonary complications (PPCs), compared with neostigmine, remains to be determined. We performed a meta-analysis of randomized controlled trials (RCTs) to compare the incidence of PPCs between patients who received sugammadex versus neostigmine.

**Methods:**

Relevant studies were obtained by searching the PubMed, Embase, and Cochrane Library databases. A random effects model incorporating the potential heterogeneity was used to pool the results.

**Results:**

Fourteen RCTs including 1478 adult patients who underwent surgeries with general anesthesia were included, and of these, 753 received sugammadex and 725 received neostigmine for reversal of NMB. The pooled results showed that sugammadex was associated with a lower risk of overall PPCs compared to neostigmine (odds ratio [OR]: 0.62, 95% confidence interval [CI]: 0.43–0.89, *p* = 0.01; *I*^2^ = 0%). This finding remained consistent after exclusion of two studies with potential overlapping events (OR: 0.58, 95% CI: 0.36–0.96, *p* = 0.03; *I*^2^=9%). Stratified analyses according to the categories of PPCs showed that sugammadex was associated with a significantly lower risk of postoperative respiratory failure (OR: 0.60, 95% CI: 0.38–0.97, *p* = 0.04; *I*^2^ = 0%) but not of postoperative pulmonary infection (OR: 0.79, *p* = 0.71), atelectasis (OR: 0.78, *p* = 0.33), or pneumothorax (OR: 0.87, *p* = 0.79).

**Conclusions:**

Compared with neostigmine, the use of sugammadex for reversal of NMB was associated with a lower risk of PPCs, mainly due to a lower incidence of postoperative respiratory failure with the use of sugammadex.

## Background

Currently, medications that provide a neuromuscular block (NMB) are routinely administered in major surgeries and procedures to facilitate airway intubation and maintain surgical status (Zafirova & Dalton, 2018; Stauble & Blobner, 2020). Although reversal agents are applied to eliminate NMB after surgery, residual NMB may remain and has been associated with an increased risk of postoperative pulmonary complications (PPCs), such as hypoxia, atelectasis, pulmonary infection, etc. (Miskovic & Lumb, 2017; Raval et al., 2020). With a varying incidence of 1–23%, PPCs have been correlated to higher short-term and long-term mortality in patients after major surgical procedures (Raval et al., 2020; Cammu, 2020). Therefore, effective strategies to reduce the incidence of residual NMB-related PPCs are important to improve the overall prognosis of patients after major surgeries.

Conventionally, neostigmine, an acetylcholinesterase inhibitor, is used as a NMB reversal drug (Haerter & Eikermann, 2016). However, accumulating evidence suggests that the NMB reversal efficacy of neostigmine is less than optimal, particular for deep NMB (Dubois & Mulier, 2013). Sugammadex, a gamma-cyclodextrin that specifically binds to rocuronium, has been shown to confer faster and more complete reversal of NMB as compared with neostigmine (Carron et al., 2016a; Hristovska et al., 2017; Hristovska et al., 2018; Won et al., 2016). Additionally, the use of sugammadex is suggested to be associated with fewer overall postoperative adverse events compared with neostigmine (Hristovska et al., 2017), leading to accelerated postoperative discharge of patients after general anesthesia (Carron et al., 2020; Carron et al., 2017). However, clinical studies comparing the incidence of PPCs after NMB reversal with sugammadex versus neostigmine have provided inconsistent results (Schaller et al., 2010; Geldner et al., 2012; Carron et al., 2013; Brueckmann et al., 2015; Koyuncu et al., 2015; Unal et al., 2015; Hakimoglu et al., 2016; Agha et al., 2017; Yagan et al., 2017; Alday et al., 2019; Claroni et al., 2019; Ba et al., 2020; Lee et al., 2020; Togioka et al., 2020). For example, some previous randomized controlled trials (RCTs) showed that sugammadex is associated with a reduced risk of PPCs as compared with neostigmine (Carron et al., 2013; Unal et al., 2015), while other studies did not show a significant difference regarding the incidence of PPCs among patients allocated to the two drugs (Schaller et al., 2010; Geldner et al., 2012; Brueckmann et al., 2015; Koyuncu et al., 2015; Hakimoglu et al., 2016; Agha et al., 2017; Yagan et al., 2017; Alday et al., 2019; Claroni et al., 2019; Ba et al., 2020; Lee et al., 2020; Togioka et al., 2020). Moreover, the outcome of PPCs was rarely observed in previous meta-analyses comparing the efficacy and safety between sugammadex and neostigmine for reversal of NMB (Carron et al., 2016a; Hristovska et al., 2017; Hristovska et al., 2018). Therefore, it remains undetermined whether sugammadex is superior to neostigmine with regard to the risk of PPCs. In view of the limited sample sizes in previous RCTs, which may cause potential statistical inadequacy, we aimed to compare the influence of NMB reversal with sugammadex or neostigmine on the risk of PPCs after general anesthesia via a meta-analysis.

## Methods

The PRISMA (Preferred Reporting Items for Systematic Reviews and Meta-Analyses) statement (Moher et al., 2009) and the Cochrane Handbook guidelines (Higgins & Green, 2011) were followed during the designing and implementation of this study.

### Search strategy

The PubMed, Embase, and Cochrane Library (Cochrane Center Register of Controlled Trials) databases were searched from inception to April 5, 2020 for relevant studies with a combined strategy of (1) "sugammadex" OR "selective relaxant binding agent" OR "SRBA" OR "org 25969" OR "bridion" and (2) "neostigmine". This extensive search strategy was used to avoid missing any potentially relevant RCTs. Only clinical studies published in English or Chinese were considered. The references of related reviews and original articles were also searched as a complementation.

### Study selection

Parallel-group RCTs published as peer-reviewed articles in English or Chinese were considered for this meta-analysis. The inclusion criteria according to the PICO principle were (1) Patients: adult patients undergoing surgeries with general anesthesia with NMB were included; (2) Intervention: sugammadex was used as intervention for NMB reversal; (3) Comparison: neostigmine was used as control for NMB reversal; and (4) Outcomes: reporting of the incidence of PPCs during the perioperative periods. PPCs were defined in accordance with previous consensus of multiple studies, which generally included respiratory failure, respiratory infection, atelectasis, pneumothorax, pleural effusion, etc. (Miskovic & Lumb, 2017; Tao et al., 2014; Jammer et al., 2015). The definition of postoperative respiratory failure was in accordance with the European Perioperative Clinical Outcome criteria, which included postoperative PaO2 < 8 kPa (60 mmHg) on room air, a PaO2:FIO2 ratio < 40 kPa (300 mmHg), or arterial oxyhemoglobin saturation measured with pulse oximetry < 90% and requiring oxygen therapy (Miskovic & Lumb, 2017). Reviews, studies including children or neonates, preclinical studies, observational studies, and repeated reports were excluded.

### Data extraction and quality assessment

The literature search, data extraction, and quality evaluation were performed by two authors independently. Any disagreement was resolved by consensus between the two authors. We extracted data regarding study information (first author, publication year, and study country), study design (blind or open-label), patient and surgery characteristics (number of participants, mean age, gender, and surgery type), agent for NMB, and dose of sugammadex or neostigmine. The primary outcome of the meta-analysis was the incidence of overall PPCs, and the secondary outcomes were the incidences of individual categories of PPCs, including postoperative respiratory failure, respiratory infection, atelectasis, pneumothorax, pleural effusion, etc.. For studies with unclear outcome data, the corresponding authors of the original studies were contacted via email for further clarification. Quality evaluation was achieved using the Cochrane’s Risk of Bias Tool (Higgins & Green, 2011), according to the following aspects (1) random sequence generation, (2) allocation concealment, (3) blinding of participants and personnel, (4) blinding of outcome assessors, (5) incomplete outcome data, (6) selective outcome reporting, and (7) other potential bias.

### Statistical analysis

The incidence of PPCs in each arm was evaluated via the odds ratio (OR) and its 95% confidence interval (CI). We used the Cochrane’s Q test to detect the heterogeneity, and significant heterogeneity was suggested if *p* < 0.10 (Higgins & Thompson, 2002). The *I*^2^ statistic was also calculated, and an *I*^2^ > 50% reflected significant heterogeneity. Pooled analyses were calculated using a random effects model, because this method incorporates the influence of potential heterogeneity and provides a more generalized result (Higgins & Green, 2011). Sensitivity analyses based on the omission of one study at a time were performed to evaluate the stability of the meta-analysis result. Stratified analyses were performed to evaluate the risk of each category of PPCs in patients in the sugammadex or neostigmine reversal group. Publication bias was evaluated by visual inspection of funnel plots and the Egger’s regression asymmetry test (Egger et al., 1997). Values of *p* < 0.05 were considered statistically significant. The RevMan (Version 5.1; Cochrane, Oxford, UK) and Stata software (Version 12.0; Stata, College Station, TX, USA) were applied for statistical analyses.

## Results

### Search results

**Fig. 1:**
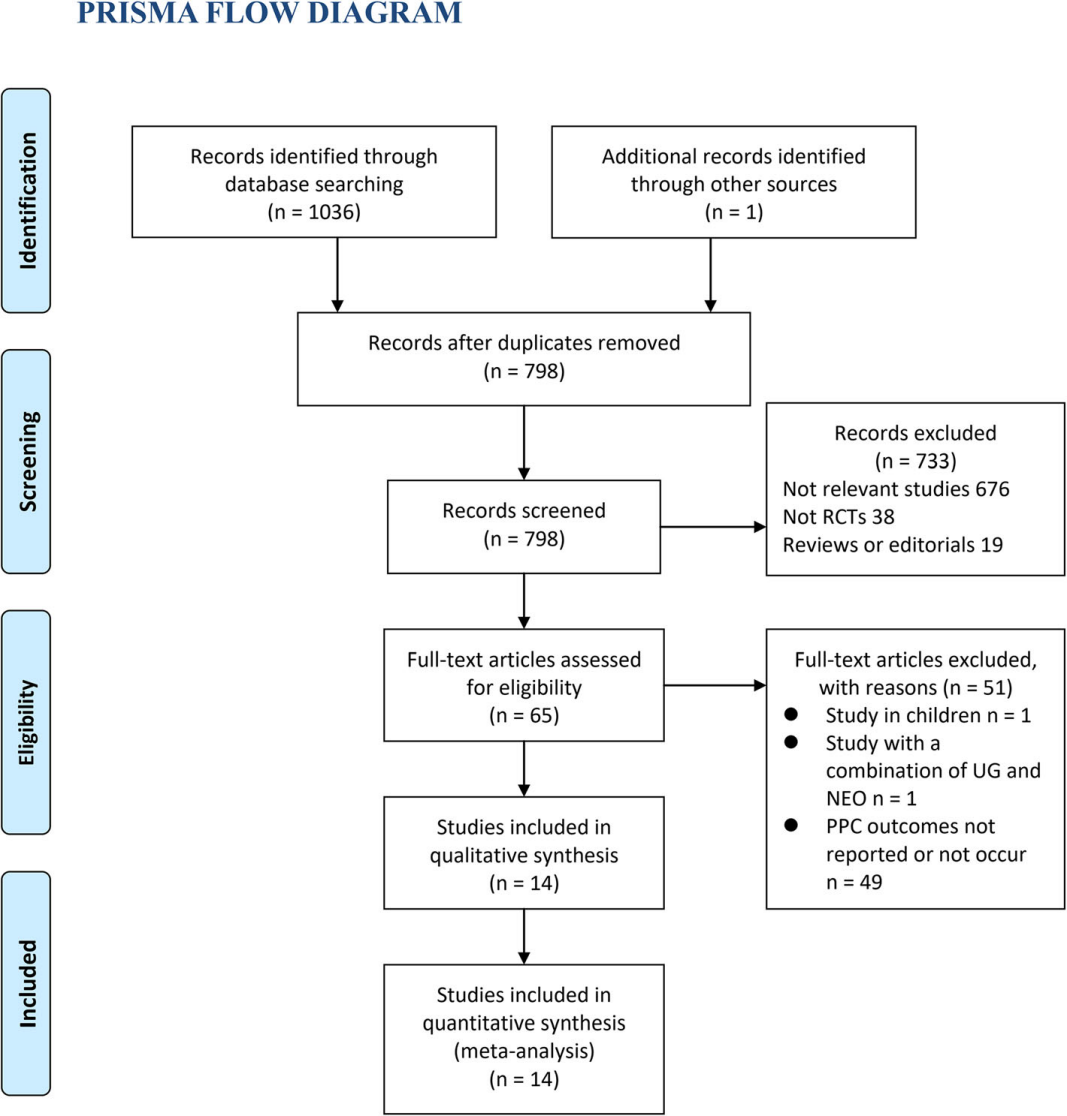
Flow chart of literature search

In total, 1037 articles were obtained through the initial database searches. After exclusion of duplicate studies, 798 articles were screened. Among them, 733 articles were subsequently excluded based on titles and abstracts, primarily because these studies were not relevant. Among the 65 potentially relevant articles, 51 were further excluded after full-text review based on reasons listed in Fig. 1. Finally, 14 RCTs were included (Schaller et al., 2010; Geldner et al., 2012; Carron et al., 2013; Brueckmann et al., 2015; Koyuncu et al., 2015; Unal et al., 2015; Hakimoglu et al., 2016; Agha et al., 2017; Yagan et al., 2017; Alday et al., 2019; Claroni et al., 2019; Ba et al., 2020; Lee et al., 2020; Togioka et al., 2020).

### Study characteristics

*Table**1* presents the characteristics of the included studies. Overall, 14 RCTs including 1478 adult patients who underwent surgeries with general anesthesia were included. Of these, 753 were given sugammadex, and 725 were given neostigmine for reversal of NMB. These studies were published between 2010 and 2020 and performed in the USA, Germany, Italy, Spain, Turkey, Malaysia, South Korea, and China. The adult patients underwent various surgeries, including major abdominal surgery, laparoscopic surgery, arthroscopic surgery, extremity surgery, thoracoscopic lung cancer resection, robot-assisted radical cystectomy for bladder cancer, and surgery for obstructive sleep apnea. The sample sizes in the RCTs varied from 40 to 200. In all of the included studies, rocuronium was used for NMB. The doses of sugammadex and neostigmine varied from 0.0625 to ~ 4 mg/kg and 5 to ~ 85 μg/kg, respectively.

**Table 1:** Characteristics of the included RCTs

Study	Country	Design	Patients and surgeries	Sample size	Mean age	Male	Neuromuscular blocking agent	Sugammadex dose	Neostigmine dose	Secondary PPCs reported^a^
					years	%				
Schaller et al., 2010	Germany	R, DB	ASA I–III, various surgery type	94	42	54	Rocuronium	0.0625, 0.125, 0.25, 0.5, or 1.0 mg/kg	5, 8, 15, 25, or 40 μg/kg	A
Geldner et al., 2012	Germany	R, DB	ASA I–III, laparoscopic surgery	133	51	31	Rocuronium	4 mg/kg	50 μg/kg	A
Carron et al., 2013	Italy	R	ASA I–III, obese patients for laparoscopic removal of adjustable gastric banding	40	43	30	Rocuronium	4 mg/kg	70 μg/kg	A
Koyuncu 2014	Turkey	R, DB	ASA I–II, extremity surgery	100	27	54	Rocuronium	2 mg/kg	70 μg/kg	A
Brueckmann et al., 2015	USA	R, DB	ASA I–III, elective laparoscopic or open abdominal surgery	151	57	60	Rocuronium	2 or 4 mg/kg	17.1–84.8 μg/kg	A, B
Unal et al., 2015	Turkey	R	ASA I–II, surgery for OSA	74	46	NR	Rocuronium	2 mg/kg	40 μg/kg	A
Hakimoglu et al., 2016	Turkey	R	ASA I–II, arthroscopic surgery	60	34	65	Rocuronium	4 mg/kg	50 μg/kg	A
Loh 2017	Malaysia	R, DB	ASA I–III, various surgery type	120	44	48	Rocuronium	2 mg/kg	20 μg/kg	A
Yagan et al., 2017	Turkey	R, SB	ASA I–II, various surgery type	98	40	65	Rocuronium	2 mg/kg	50 μg/kg	A
Alday et al., 2019	Spain	R, SB	ASA I–IV, major abdominal surgery	126	68	51	Rocuronium	4 mg/kg	40 μg/kg	A, B, C
Claroni et al., 2019	Italy	R, DB	ASA I–III, robot-assisted radical cystectomy for bladder cancer	109	62	83	Rocuronium	2 mg/kg	40 μg/kg	A
Togioka et al., 2020	USA	R, OL	ASA I–IV, various surgery type	200	75	46	Rocuronium	2 mg/kg	70 μg/kg	A, B, C, D
Lee et al., 2020	South Korea	R, SB	ASA I–II, elective laparoscopic cholecystectomy	73	56	48	Rocuronium	4 mg/kg	40 μg/kg	A
Ba et al., 2020	China	R	ASA I–II, radical resection of lung cancer under thoracoscope	100	50	51	Rocuronium	2 mg/kg	2 mg	A

*RCT*randomized controlled trial; *R* randomized; *DB* double-blind; *SB* single-blind; *OL* open-label; *ASA* American Society of Anesthesiology; *OSA* obstructive sleep apnea.

^a^Indicators for secondary PPCs: A, respiratory failure; B, respiratory infection; C, atelectasis; and D, pneumothorax

### Data quality

**Fig. 2:**
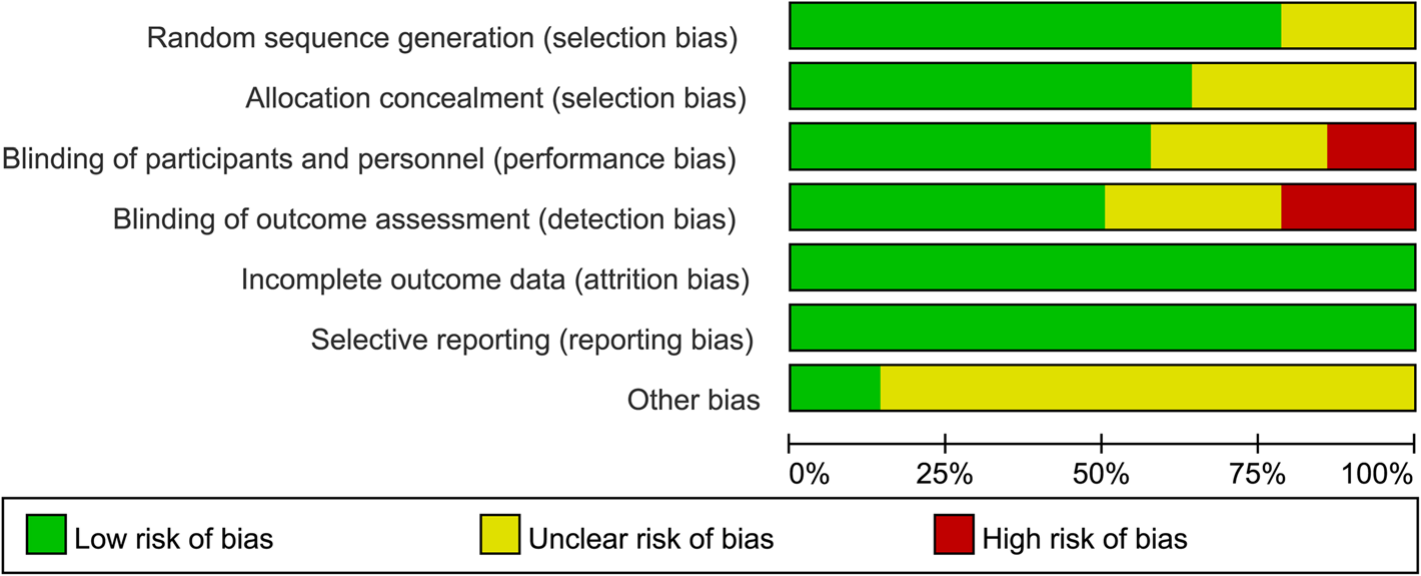
Summary of study quality evaluation using Cochrane’s risk of bias tool

Table 2 and Fig. 2 show the details of the study quality evaluation. Six of the included RCTs were double blind (Schaller et al., 2010; Geldner et al., 2012; Brueckmann et al., 2015; Koyuncu et al., 2015; Agha et al., 2017; Claroni et al., 2019), three were single blind (Yagan et al., 2017; Alday et al., 2019; Lee et al., 2020), and the rest did not apply blinding. Methods of random sequence generation were reported in 11 studies (Schaller et al., 2010; Geldner et al., 2012; Carron et al., 2013; Brueckmann et al., 2015; Hakimoglu et al., 2016; Agha et al., 2017; Yagan et al., 2017; Claroni et al., 2019; Ba et al., 2020; Lee et al., 2020; Togioka et al., 2020), and information for allocation concealment was reported in 9 studies (Schaller et al., 2010; Carron et al., 2013; Brueckmann et al., 2015; Unal et al., 2015; Agha et al., 2017; Yagan et al., 2017; Claroni et al., 2019; Lee et al., 2020; Togioka et al., 2020). The overall quality scores varied from 3 to 7.

**Table 2:** Quality evaluation with Cochrane’s risk of bias tool

	Sequence generation	Allocation concealment	Blinding of participants and personnel	Blinding of outcome assessment	Incomplete outcome data	Selective outcome reporting	Other potential threats	Total
Schaller et al., 2010	Low	Low	Low	Low	Low	Low	Unclear	6
Geldner et al., 2012	Low	Unclear	Low	Low	Low	Low	Unclear	5
Carron et al., 2013	Low	Low	Unclear	Unclear	Low	Low	Unclear	4
Koyuncu 2014	Unclear	Unclear	Low	Low	Low	Low	Unclear	4
Brueckmann et al., 2015	Low	Low	Low	Low	Low	Low	Low	7
Unal et al., 2015	Unclear	Low	Unclear	Unclear	Low	Low	Unclear	3
Hakimoglu et al., 2016	Low	Unclear	Unclear	Unclear	Low	Low	Unclear	3
Loh 2017	Low	Low	Low	Low	Low	Low	Unclear	6
Yagan et al., 2017	Low	Low	Low	High	Low	Low	Unclear	5
Alday et al., 2019	Unclear	Unclear	Low	High	Low	Low	Unclear	3
Claroni et al., 2019	Low	Low	Low	Low	Low	Low	Unclear	6
Togioka et al., 2020	Low	Low	High	High	Low	Low	Low	4
Lee et al., 2020	Low	Low	High	Low	Low	Low	Unclear	5
Ba et al., 2020	Low	Unclear	Unclear	Unclear	Low	Low	Unclear	3

### Meta-analysis results

Two studies reported the incidence rates for individual categories of PPCs rather than the total number of patients with PPCs in each arm (Brueckmann et al., 2015; Alday et al., 2019). For these studies, potential overlapping of the PPC events could have occurred in the same patients. We contacted the corresponding authors for further clarification. However, no response was received. For these two studies (Brueckmann et al., 2015; Alday et al., 2019), we first simply added the numbers of each category of PPC as the overall number of PPCs and included these totals in the main meta-analysis; then, sensitivity analysis with exclusion of these two studies with patients who experienced potentially overlapping PPCs were performed.

**Fig. 3:**
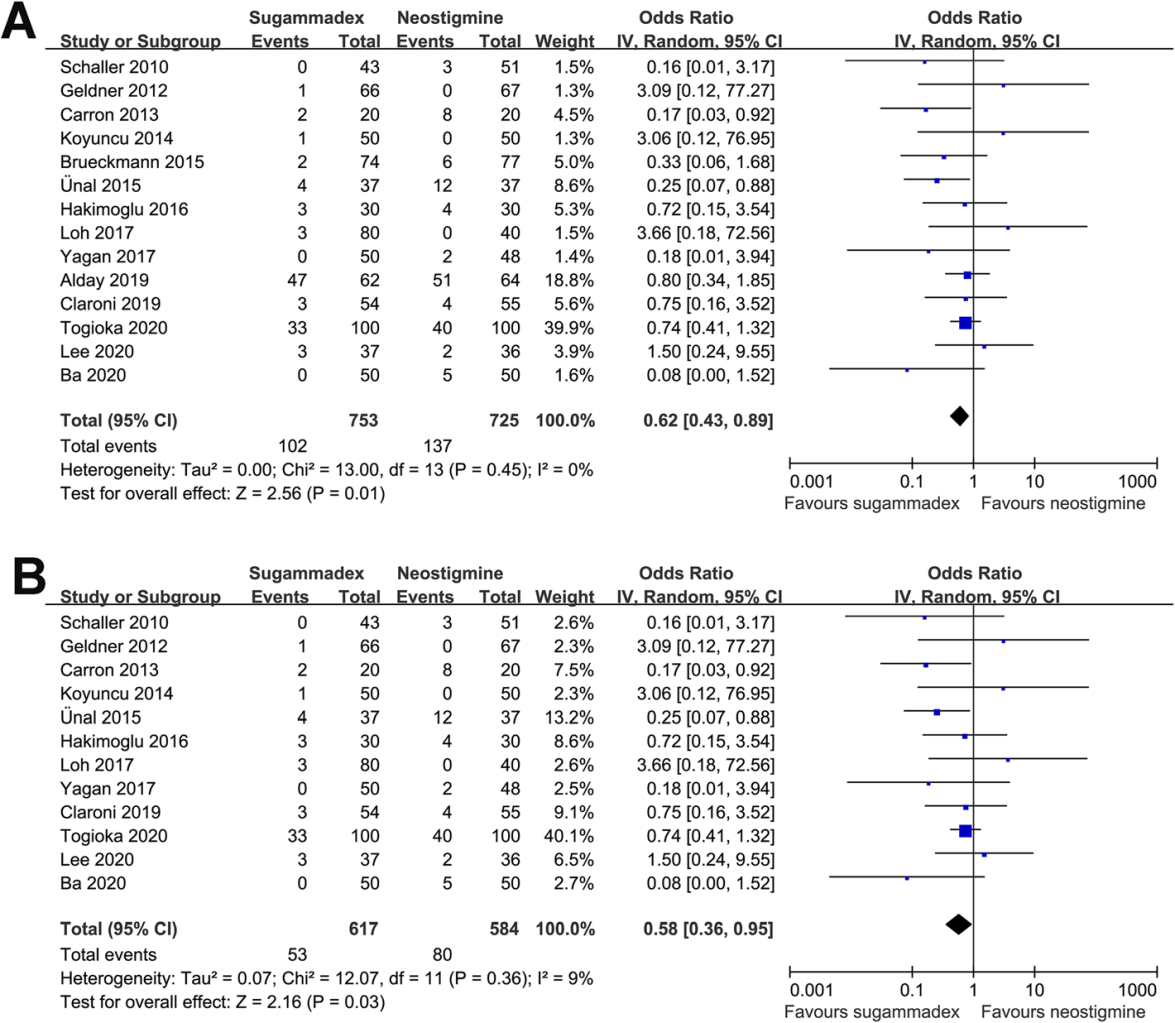
Forest plots for the meta-analysis comparing sugammadex and neostigmine for the incidence of PPCs. **A** Main meta-analysis and **B** sensitivity analysis based on exclusion of two studies with patients who potentially had overlapping PPCs

No significant heterogeneity was detected for the main meta-analysis (*p* for Cochrane’s Q test = 0.45, *I*^2^ = 0%). The pooled results with a random effects model showed that sugammadex was associated with a significantly lower risk of overall PPCs compared with neostigmine (14 studies; OR: 0.62, 95% CI: 0.43–0.89, *p* = 0.01; Fig. 3A). Sensitivity analyses with omission of one study at a time showed similar results (OR: 0.55–0.68, all *p* < 0.05). Additionally, exclusion of the two studies including patients with potentially overlapping PPCs did not significantly change the results (12 studies; OR: 0.58, 9% CI: 0.36–0.96, *p* = 0.03; I^2^ = 9%; Fig. 3B).

**Fig. 4:**
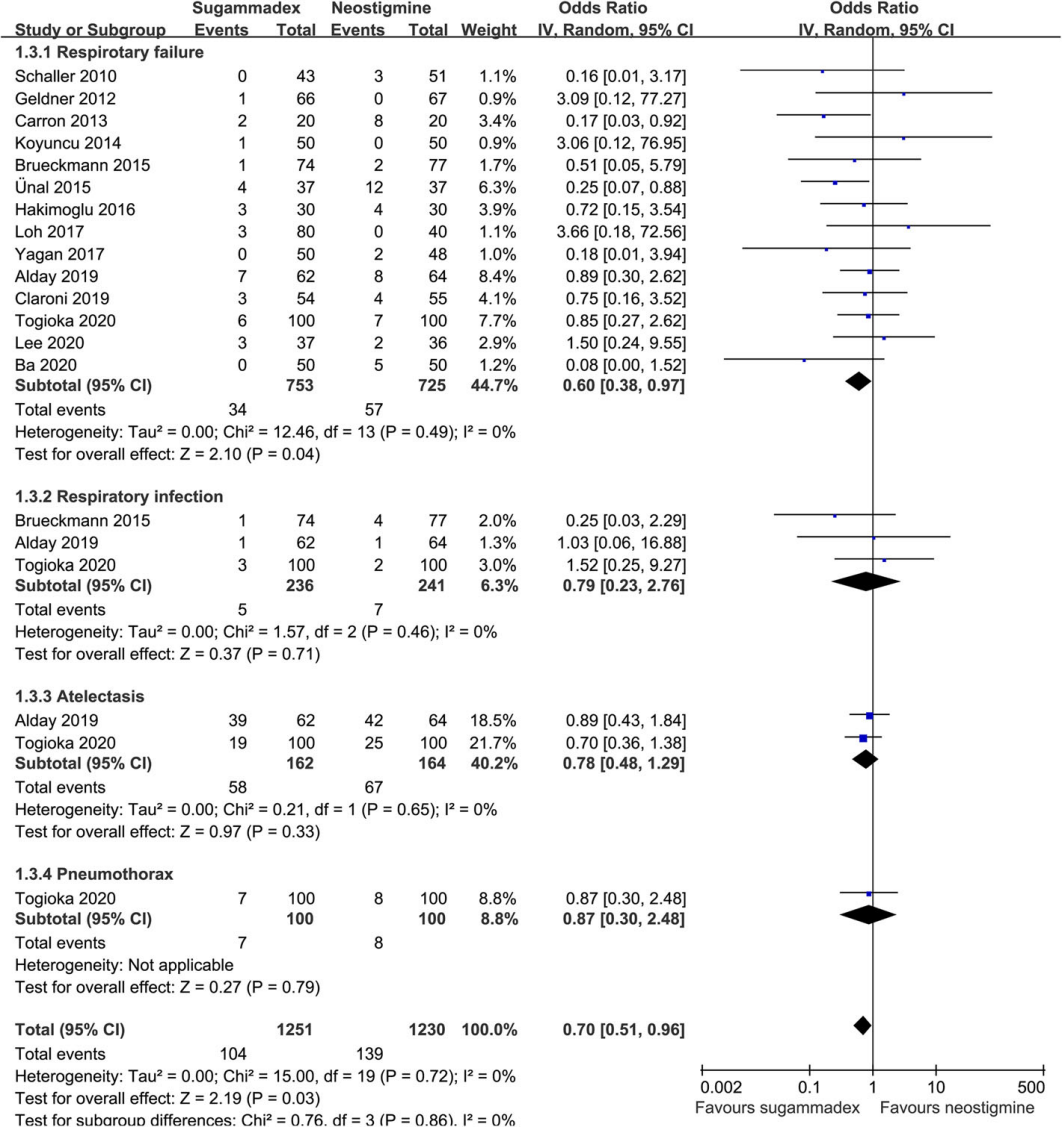
Stratified analyses according to the individual categories of PPCs

Stratified analyses according to the categories of PPCs showed that sugammadex was associated with a significantly lower risk of postoperative respiratory failure (14 studies; OR: 0.60, 95% CI: 0.38–0.97, *p* = 0.04; *I*^2^ = 0%; Fig. 4) but not of postoperative respiratory infection (3 studies; OR: 0.79, *p* = 0.71), atelectasis (2 studies; OR: 0.78, *p* = 0.33), or pneumothorax (1 study; OR: 0.87, *p* = 0.79).

### Publication bias

**Fig. 5:**
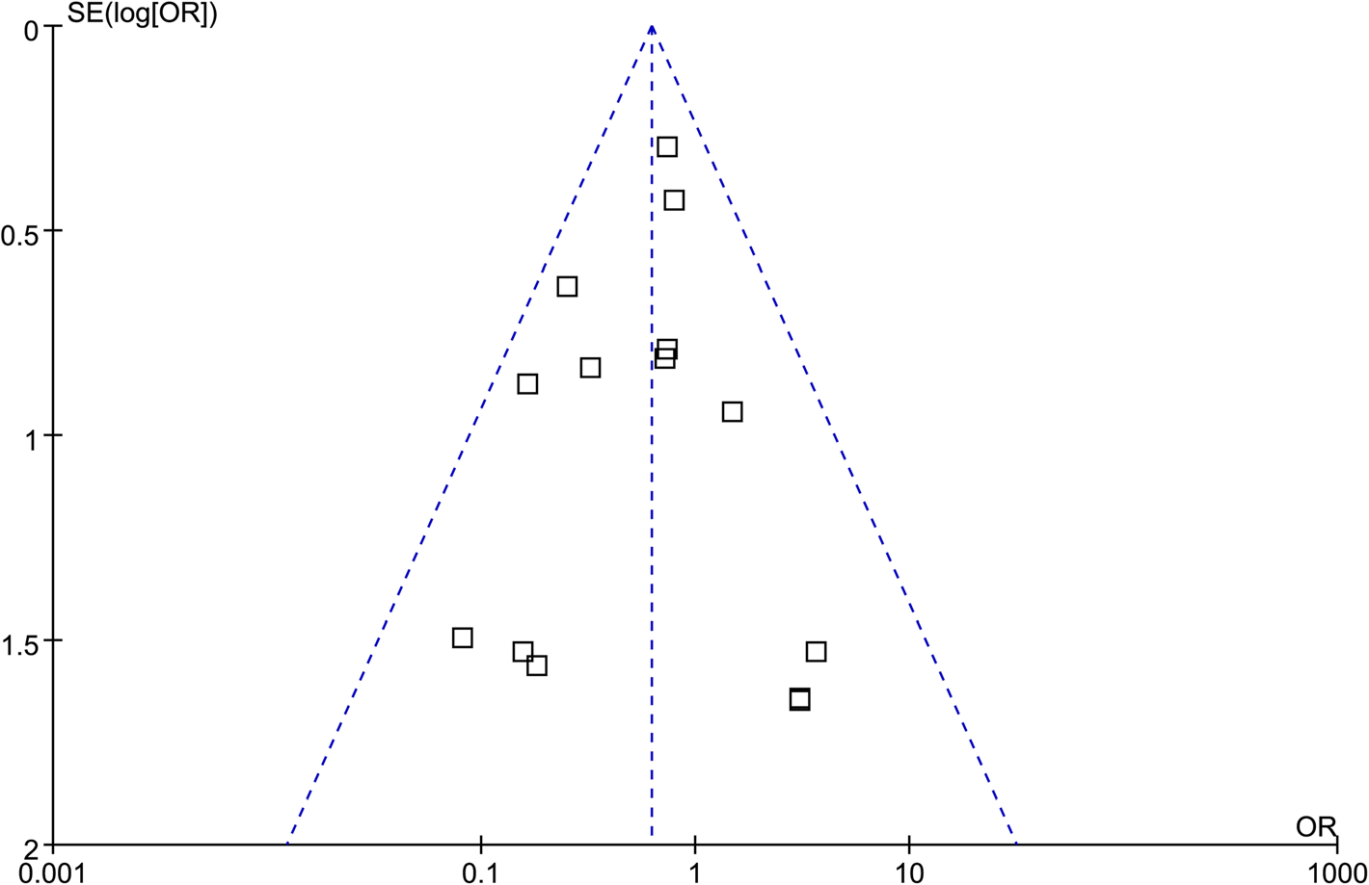
Funnel plots for the meta-analysis comparing the incidence of PPCs after the use of sugammadex and neostigmine for NMB reversal. Each square represents one of the included studies, and the plots were formed based on the data of OR (x-axis) and standard error of lg(OR) (y-axis). A symmetrical of the plots could be indicated if the distributions of the squares were symmetrical according to the vertical line as the axis

The funnel plots were symmetrical, suggesting low risk of publication bias (Fig. 5). Egger’s regression tests showed similar results (*p* = 0.65).

## Discussion

In this meta-analysis of 14 RCTs, we found that compared with the conventional NMB reversal drug neostigmine, sugammadex was associated with a significantly lower risk of overall PPCs. Stratified analyses showed that patients who received sugammadex had a significantly reduced risk of postoperative respiratory failure compared with those who received neostigmine, while the incidences of other PPCs, including pulmonary infection, atelectasis, and pneumothorax, did not differ significantly between the groups. These findings suggest that NMB reversal with sugammadex was superior to that with neostigmine in providing lower incidences of overall PPCs and postoperative respiratory failure.

To the best of our knowledge, our study is the first meta-analysis specifically investigating the potential influences of NMB reversal by sugammadex or neostigmine on the incidence of PPCs in patients after general surgery. We found that sugammadex was effective at reducing overall PPCs compared with neostigmine. These results were further confirmed by sensitivity analyses, while stratified analyses suggested the benefit of sugammadex for PPCs as compared to neostigmine was mainly driven by the lower risk of postoperative respiratory failure. Interestingly, 12 of the included RCTs did not show a significant difference regarding the incidence of PPCs between patients who received sugammadex versus neostigmine (Schaller et al., 2010; Geldner et al., 2012; Brueckmann et al., 2015; Koyuncu et al., 2015; Hakimoglu et al., 2016; Agha et al., 2017; Yagan et al., 2017; Alday et al., 2019; Claroni et al., 2019; Ba et al., 2020; Lee et al., 2020; Togioka et al., 2020), which may indicate the general inadequacy of the statistical power in these RCTs to detect a significant effect on the PPC incidence. Although many factors have been related to the incidence of PPCs, residual NMB has been suggested to be one of the major determinants for the pathogenesis of PPCs (Cammu, 2020). Cohort studies have confirmed clear associations between residual NMB and the risks of various types of PPCs, such as upper airway obstruction, hypoxemia, atelectasis, and pneumonia (Bulka et al., 2016; Murphy et al., 2004; Murphy et al., 2008; Stawicki & Gessner, 2018). Furthermore, it has been suggested that even slight residual NMB may cause pharyngeal and laryngeal dysfunction and depress pulmonary ventilation, all of which might induce PPCs (De Troyer & Bastenier-Geens, 1979; Cedborg et al., 2014; Fuchs-Buder et al., 2016). Therefore, in view of the importance of residual NMB in the pathogenesis of PPCs, our finding suggested that sugammadex was superior to neostigmine for reduced PPCs and may reflect the faster and more complete NMB reversal efficacy of sugammadex compared with neostigmine (Hristovska et al., 2017). Moreover, the differences in pharmacology between sugammadex and neostigmine may also explain their different influences on PPCs. High-dose neostigmine may cause bronchospasm (Hazizaj & Hatija, 2006; Ishii et al., 2012), which may therefore adversely affect the pulmonary ventilation of the patients. Additionally, neostigmine administration after adequate reversal of NMB could lead to functional disorders of the genioglossus muscle and diaphragm, which may cause obstruction of the upper airway and respiratory failure (Eikermann et al., 2008). On the contrary, by selective binding to rocuronium, sugammadex does not affect the genioglossus muscle or diaphragmatic function (Eikermann et al., 2008; Herbstreit et al., 2010). An early study showed that sugammadex (2 and 4 mg/kg) was well tolerated and effective in patients with a history of pulmonary disease (Amao et al., 2012). Furthermore, a recent retrospective cohort study showed that in patients with chronic obstructive pulmonary disease who underwent abdominal surgery, the incidence of PPCs was lower when sugammadex was applied for NMB reversal (Park et al., 2020). The results of our present meta-analysis are consistent with the findings of the above studies, demonstrating that NMB reversal with sugammadex is associated with fewer PPCs compared with neostigmine, and sugammadex may be superior to neostigmine for patients at high risk for the development of PPCs.

It has to be mentioned that although the OR is consistent with a benefit associated with sugammadex over neostigmine for the reduced risk of respiratory failure, the number needed to treat in order to prevent one case of PPC is relatively high (NNT = 29.9) according to the pooled results of the included studies. However, prevention of PPCs is an additional benefit of sugammadex over neostigmine besides its main efficacy on the reversal of NMB. Besides, accumulating studies have shown that replacement of neostigmine with sugammadex for NMB reversal is associated with significantly reduced costs for NBM management (Carron et al., 2016b; Ren et al., 2020), which also supports the use of sugammadex in this clinical scenario.

There are some limitations in our studies. First, although no statistical heterogeneity was detected among the included studies, clinical heterogeneity may exist regarding the differences in patient characteristics, surgical type, and regimens of sugammadex and neostigmine administration among the included RCTs. Moreover, we were unable to evaluate the potential influences of the above factors on the outcomes of the meta-analysis, since stratified analyses were rarely reported in the original RCTs and no access to individual patient data was obtained. Therefore, the influences of these clinical factors on the relative influences of sugammadex compared with neostigmine on PPCs or respiratory failure remains unknown. From this perspective, a meta-analysis based on individual patient data rather than that based on the study-level data is more meaningful for clinical practice. To the best of our knowledge, no studies have attempted to develop a preoperative risk assessment tool for the determination of using sugammadex or neostigmine for reversal of NMB, and none of the included studies employed such a screening tool. Therefore, further studies are needed to determine whether the potential benefits of sugammadex over neostigmine for reducing PPCs are consistent in patients with different comorbidities and in studies with different surgical procedures. Finally, as mentioned before, the superiority of sugammadex to neostigmine for reducing overall PPCs was mainly driven by data regarding postoperative respiratory failure. For other types of PPCs, including respiratory infection, atelectasis, and pneumothorax, only 1–3 studies were included. Therefore, the non-significant results in our meta-analysis for these outcomes should be interpreted with caution, and more large-scale RCTs are needed.

In conclusion, the results of this meta-analysis based on 14 RCTs indicated that compared with neostigmine, sugammadex for reversal of NMB was associated with a lower risk of PPCs, mainly due to a lower incidence of postoperative respiratory failure after the use of sugammadex. These results may be attributed to the more rapid and complete NMB reversal achieved by sugammadex compared with neostigmine.

## Data Availability

All data generated or analyzed during this study are included in this published article.

## Abbreviations

NMB: Neuromuscular block
PPCs: Pulmonary complications
RCTs: Randomized controlled trials
PRISMA: Preferred reporting items for systematic reviews and meta-analyses
OR: Odds ratio
CI: Confidence interval
